# An evaluation of a tailored intervention on village doctors use of electronic health records

**DOI:** 10.1186/1472-6963-14-217

**Published:** 2014-05-14

**Authors:** Peiyuan He, Zhaokang Yuan, Yong Liu, Guoqing Li, Huanhuan Lv, Ji Yu, Mark F Harris

**Affiliations:** 1 School of Public Health, Nanchang University, 461 Baiyi Road, Nanchang, Jiangxi 330006, China; 2 Rural Health Office of Jiangxi Provincial Health Bureau, 1 South 1st Road in Courtyard of Jiangxi Provincial government, Nanchang, Jiangxi 330000, China; 3 Centre for Primary Health Care and Equity, University of New South Wales, Sydney, NSW 2052, Australia

**Keywords:** EHR, Village doctors, Pilot study, Personalized intervention, Rural areas

## Abstract

**Background:**

To describe and evaluate the effectiveness of tailored intervention on village doctor’s use of electronic health records (EHR) in rural community health services in less developed areas.

**Methods:**

Ten townships were selected. In each township, two similar health service station (CHSS) were chosen. One was randomly as allocated to the intervention group, the other to the control group. Over six monthly visits, a structured on-site intervention including education, supervision and technical support was provided to village doctors in the intervention group tailored to their needs. The Control group received no visits. A sample of 20 families from each CHSS was randomly chosen. An online evaluation of each family’s EHR was conducted by the investigators at baseline and at the end of the 6 month intervention.

**Results:**

In the intervention group, the proportion of households with complete records increased: basic personal information from 2.6% to 32.5%, (Z = -15.099, P = 0.000) and health education records from 0.3% to 1.6% (Z = -4.459, P = 0.000). Similarly at baseline none of the 80 elders had her records. This increased in the intervention group to 16.4% recorded in part and 37.0% in full (Z = -7.480, P = 0.000). The proportion of complete health management records for children aged 1 to 2 years and 3 to 6 years increased from 28.6% and 33.3% to 66.7% and 74.2% respectively (the difference of children group 3 to 6 years of age was statistically significant, Z = -3.860, p = 0.000). The proportion of complete basic clinic records in the intervention group increased from 7.6% to 13.9% (Z = -3.252, P = 0.001). There were no significant differences in the control group.

**Conclusions:**

The pilot study showed that a on-site education, supervision and technical support tailored to their needs was associated with improvements in village doctors use of EHR. This model is worthy of implementation in other rural areas.

## Background

Health information technology has a key role in the deepening medical health system reform in China. The construction of a regional health information platform based on the electronic health record (EHR) is an important prerequisite for this [1]. EHR is an electronic, standardized, scientific record of a resident’s health management (including disease prevention, health protection, health education and clinical care). It is an information resource that collects information from multiple channels to track the health of a resident throughout the life course, covering a variety of health-related actions [2]. In 2009 the Chinese National Ministry of Health launched a nationwide EHR program. The plan was for the coverage rate of EHR to reach 30% of the population in rural areas and 50% in urban areas by 2011. By 2020 the aim was for a uniform, effective, high quality EHR system to have been established, maintained and used in health services for the residents in both urban and rural areas [3]. So far, the coverage rate for the EHR in China has achieved national goals. However the use of the EHR is not satisfactory [4]. Especially in less developed rural areas, doctors are often unwilling to use EHR due to lack of understanding of its importance and skill in its operation [4,5]. The huge investment of government to the construction of EHR system has not yet achieved the desired results [6].

With the funding support of the China-Australia Health and HIV/AIDS Facility (CAHHF, HSS403), the project team carried out a pilot study of intervention to increase EHR use by village doctors in Chongyi County, a less developed rural area. The intervention included supervision, technical support and education. This article evaluates the effect of this intervention using data extracted from the online EHR to provide a basis for further implementation of the EHR in rural areas.

## Methods

### Study population and sample

Ten of the 15 townships in Chongyi County were chosen as research sites, on the basis of their population. Two adjacent similar Community Health Service Stations (CHSS) were chosen from each of these 10 townships. These pairs were matched based on the education and training, age and service population of the village doctors. One of them was randomly allocated to the intervention group and the other to the control group. In each CHSS, by simple random sampling, 20 families’ EHR from each CHSS were selected for evaluation.

### Intervention

The project team carried out a six monthly visits to CHSS in intervention group. The team comprised public health experts, software experts, and County health bureau officials. The intervention included three aspects: 1. Supervision and checking the quality of village doctor’s use of the EHR including data entry and retrieval of information for follow up care 2. Technical support about how to use EHR tailored to the village doctors circumstances and needs including solving problems that they encountered in the process of using EHR. 3. Face to face education about EHR policies and benefits, including hands on training in the proactive and timely use of EHR. The control group did not receive the intervention but was observed in parallel.

### Data collection

The baseline survey was conducted in October 2011. A sample of 20 families from each CHSS was randomly assigned (a total of 400 families) and an evaluation of each family’s on-line EHR was conducted by the investigators. The information on these family members (at baseline 1451 residents, at follow up 1467 residents) was collected online using a structured data collection form by investigators. The final evaluation was conducted in March 2012. The School of Public Health, Nanchang University was responsible for the formulation of the data collection form, and the training of the investigators. The investigators were postgraduate students from the School of Public health, Nanchang University.

The data collection form determined the completeness of records in the EHR of the following: basic personal information, health examinations, health education, vaccinations, child health management, management of the elderly and basic clinic care. Evaluation of these indicators was based on integrity of the case file records at time of last entry. If the record was 80% complete or higher, we classified its as ‘full complete’. If it was between 1 and 80%, we classified it as ‘part complete’. If there was no information in the record, we classified it as ‘absent’.

### Quality control

Project members and postgraduates were trained to undertake the survey. They received help from the staff of Chongyi Health Bureau to increase the cooperation of village doctors. Survey data was input into a computer data base by specially assigned person and checked for errors.

### Statistics analysis

SPSS19.0 was used to sort and analyze the data. Chi-square tests were used to estimate the difference of gender and age of respondents before and after the intervention. Two–sample *t*-test was used to estimate the difference of age of village doctors between intervention and control group. Mann–Whitney *U* test was conducted to analyze change in the completeness of the public health and basic clinic records. A two-sided significance level was set at <0.05 for all tests.

### Ethics

The study was approved by the Human Research Ethics Committee of Nanchang University.

## Results

### Demographic analysis

Each CHSS has a least one village doctor in Chongyi County. There were 9 male doctors and 1 female doctor in both the intervention and control groups. The average age of doctors was 39.0 and 38.7 years respectively. There were no significant differences in and age (t = -0.117, P = 0.910) between intervention and control groups. All village doctors had secondary school qualifications as their highest educational attainment.

The demographic characteristics of residents are shown in Table 1. Before the intervention, 736 and 715 residents were selected, with an average age of 36.9 and 36.8 years old in intervention and control groups. After the intervention, 736 and 731 residents were selected, with an average age of 36.2 and 36.5 years old respectively. Before and after the experiment there were no significant differences in gender between the intervention and control groups (Table 1).

**Table 1:** Respondents before and after the intervention

**Index**	**Intervention group**				***χ***^**2**^	**P**	**Control group**				***χ***^**2**^	**P**
	**Before (736)**		**After (736)**				**Before (715)**		**After (731)**			
	**n**_**1**_	**%**	**n**_**2**_	**%**			**n**_**3**_	**%**	**n**_**4**_	**%**		
Gender*												
Male	378	51.4	382	51.9	0.044	0.835	381	53.3	386	52.8	0.034	0.845
Female	358	48.6	354	48.1			334	46.7	345	47.2		
Age (year)**												
<20	174	24.3	176	23.9	0.442	0.931	150	21.0	144	19.8	0.284	0.970
20~	249	33.8	238	32.3			259	36.2	270	36.5		
40~	206	28.0	212	28.8			220	30.8	217	30.2		
60~	107	13.9	110	14.9			86	12.0	100	13.5		

*Gender between intervention and control group before the intervention, *χ*^2^ = 0.540, P = 0.462 and after the intervention, *χ*^2^ = 0120, P = 0.729.

**Age between intervention and control group before the intervention, *χ*^2^ = 4.417, P = 0.220 and after the intervention, *χ*^2^ = 5.733, P = 0.125.

### The EHR in general population

In the intervention group, the proportion of individual residents records with complete basic personal information increased from 2.6% to 32.5% after the intervention (Z = -15.099, P = 0.000). In the control group, there no change (0.0% to 0.5%; Z = -2.536, P = 0.011). In the intervention group, the proportion of complete health examination records increased from 30.5% to 46.9% (Z = -3.574, P = 0.000). In the control group, it also increased from 20.3% to 38.2%, (Z = -3.574, P = 0.000). In the intervention group, the proportion of complete health education records increased slightly from 0.3% to 1.6%. In control group, there were no fully complete health education records before or after the intervention.

In intervention group, the proportion of complete residents’ basic clinic records increased from 7.6% to 13.9% (Z = -3.252, P = 0.001). In control group, there no significant change (8.5% to 10.3%, Z = -1.009, P = 0.313) (Table 2).

**Table 2:** Basic personal information, health examination, health education and basic clinic records before and after the experiment in EHR

**Index**	**Intervention group**				**Z**	**P**	**Control group**				**Z**	**P**
	**Before (736)**		**After (736)**				**Before (715)**		**After (731)**			
	**n**_**1**_	**%**	**n**_**2**_	**%**			**n**_**3**_	**%**	**n**_**4**_	**%**		
Basic personal information												
No	1	0.1	0	0.0	-15.099	<0.001	5	0.7	1	0.1	-2.536	0.011
Part	716	97.3	497	67.5			710	99.3	726	99.4		
Full	19	2.6	239	32.5			0	0.0	4	0.5		
Health examination												
No	393	53.3	373	50.7	-3.574	<0.001	417	58.3	361	49.4	-5.319	<0.001
Part	118	16.1	18	2.4			153	21.4	91	12.4		
Full	224	30.50	345	46.9			145	20.3	279	38.2		
Health education												
No	734	99.7	711	96.6	-4.459	<0.001	714	99.9	729	99.7	-0.599	0.576
Part	0	0.0	13	1.8			1	0.1	2	0.3		
Full	2	0.3	12	1.6			0	0.0	0	0.0		
Basic clinic records												
No	669	90.9	630	85.6	-3.252	0.001	636	89.0	638	87.3	-1.009	0.313
Part	11	1.5	4	0.5			18	2.5	18	2.5		
Full	56	7.6	102	13.9			61	8.5	75	10.3		

### Vaccination and health management records of children

In intervention group, before and after the intervention the proportion of complete child vaccination records increased from 15.4% to 33.3%, (Z = -1.943, P = 0.052). In control group, the proportion did not change (18.6% to 17.5%, Z = -0.388, P = 0.698) (Table 3).

**Table 3:** Vaccination records before and after the experiment in EHR

**Index**	**Intervention group**				**Z**	**P**	**Control group**				**Z**	**P**
	**Before (26)**		**After (45)**				**Before (43)**		**After (57)**			
	**n**_**1**_	**%**	**n**_**2**_	**%**			**n**_**3**_	**%**	**n**_**4**_	**%**		
Vaccinate												
No	22	84.6	30	66.7	-1.943	0.052	35	81.4	44	77.2	-0.388	0.698
Part	0	0.0	0	0.0			0	0.0	3	5.3		
Full	4	15.4	15	33.3			8	18.6	10	17.5		

In intervention group, before and after the intervention the proportion of complete health management records for children aged 3 to 6 years increased from 33.3% to 74.2%, (Z = -3.860, P = 0.000). In control group, the proportion of health management records of three children did not change significantly (Table 4).

**Table 4:** Management records of children before and after the experiment in EHR

**Age**	**Intervention group**				**Z**	**P**	**Control group**				**Z**	**P**
	**Before (26)**		**After (45)**				**Before (43)**		**After (47)**			
	**n**_**1**_	**%**	**n**_**2**_	**%**			**n**_**3**_	**%**	**n**_**4**_	**%**		
<1 year												
No	6	85.7	0	0.0	-	-	0	0.0	1	25.0	-0.500	0.800
Part	1	14.3	0	0.0			0	0.0	0	0.0		
Full	0	0.0	2	100.0			1	100.0	3	75.0		
1 ~ 2 years												
No	5	71.4	3	25.0	-1.769	0.120	5	41.7	4	28.6	-0.584	0.595
Part	0	0.0	1	8.3			2	16.6	3	21.4		
Full	2	28.6	8	66.7			5	41.7	7	50.0		
3 ~ 6 years												
No	10	55.6	5	16.1	-3.860	<0.001	11	36.7	12	32.4	-0.248	0.804
Part	2	11.1	3	9.7			11	36.7	15	40.5		
Full	6	33.3	23	74.2			8	26.6	10	27.1		

### Health management of the elderly

There were significant increases in the completeness of records of health management of the elderly in both the intervention and control groups, before and after the intervention (Table 5).

**Table 5:** Health management records of the elderly before and after the intervention

**Index**	**Intervention group**				**Z**	**P**	**Control group**				**Z**	**P**
	**Before (80)**		**After (73)**				**Before (69)**		**After (72)**			
	**n**_**1**_	**%**	**n**_**2**_	**%**			**n**_**3**_	**%**	**n**_**4**_	**%**		
No	80	100.0	34	46.6	-7.480	<0.001	68	98.6	28	38.9	-7.404	<0.001
Part	0	0.0	12	16.4			1	1.4	16	22.2		
Full	0	0.0	27	37.0			0	0.0	28	38.9		

## Discussion

The EHR is a tool which may contribute to the quality of the community residents’ healthcare. By using the EHR, primary health care providers can better understand the changing health status of their communities, track and respond to trends in illness and provide timely and appropriate interventions [7]. Also, the EHR can help doctors review patient’s condition, contributing to better diagnosis and consistent treatment of the disease. Complete and accurate electronic health records can improve the efficiency and quality of clinical practice [8]. A study in Washington, USA found that use of the EHR could save 40% of health care practitioners’ time compared with paper records to get the same information [9]. At present, in developed countries such as Australia, Netherlands and UK, the coverage rates of EHR are over 90%, but the use and sharing of records between services is not generally high [10-13]. China faces a similar challenge [14].

There may be multiple reasons why medical staff in China are reluctant to use the EHR [4,14,15]. Three are especially important for rural doctors. The first is that many older doctors lack the computer knowledge and skill to operate the EHR system effectively. The second is that doctors are often unaware of the assistance that EHR can provide in improving the quality and efficiency of their work. The third is that, when doctors record all health service information into EHR, this exposes their own practices to greater scrutiny by other doctors and government officials [16]. These are difficult but important problems to address. In less developed areas, the research on the EHR has been focused on the knowledge, attitudes and satisfaction of medical personnel and patients with EHR. There are only a limited number of descriptive of her use in China. Most of this did not evaluate use before and after an intervention and involve a control group [15,17,18]. This study extends and complements previous research.

### Public health records

In our study, training and support of village doctors in the use of the EHR was associated with improved recording of basic health information, health examination, health education, vaccination, child health management and elderly health care. In the intervention program, project team members explained the basic public health services policy and funding programs to village doctors in order to help them understand the importance of public health work in rural areas, provided technical guidance to them using EHR to record public health services, removed barriers for village doctors carrying out the EHR work and motivated them in the use of the EHR. A descriptive survey of EHR use in five counties in Henan province showed that the proportion of complete basic personal information and vaccinate records were 5.1% and 18.3% respectively [19]. These are similar to the baseline results of this study. After the intervention, the proportion of complete basic personal information and vaccinate records increased significantly in intervention group.

### Health examination and the elder health management

Health examination and the elderly health management records improved both in intervention group and control group. This may have been caused by another Chongyi county government program which regularly organized residents to have physical examinations and arranged for the results to be entered into the EHR.

### Basic clinic records

There was some improvement in basic clinic records in the intervention group but not in control group. At present, due to limited national investment in public health, private village clinics (CHSS) rely on patient payments as their main source of income. After the implementation of national essential drug system but before subsidies for no profit on the sale drugs, the vast majority of village doctors’ medical income came from the sale of drugs. This left village doctors using EHR to carry out basic medical services in a conflicted situation. On the one hand, they wanted to make full use of the EHR as an effective tool to carry out routine medical work, thereby improving the operational level and the quality of work. On the other hand, if complete, comprehensive and standardized basic clinic information was updated into EHR, their medical business practices would be exposed to scrutiny by other village doctors, medical experts at the county level, and government administrators. In such a contradictory situation, the vast majority of village doctors chose to not use the EHR to comprehensively record basic medical services. Our intervention was able to overcome some but not all of these problems by demonstrating how the net effect of using the EHR could be positive.

### Strengths and limitations

The gender and age of residents and doctors in the intervention and control groups before and after the intervention were similar and did not change significantly. The two groups of adjacent CHSS were also similar with respect to their economic and social conditions and the doctors’ service population reducing the confounding effects of these on the comparative analysis between the intervention and control groups.

This study was conducted with a relatively small sample of households in one rural county and thus the findings should be generalized to other areas with caution. Only a limited number of aspects of the recording of public health and general medical service provision could be examined in this study. It would be useful to replicate the study in other rural areas, possibly using a cluster randomized controlled trial methodology.

## Conclusion

In association with a structured intervention conducted with village doctors to support great use of the EHR, the completeness of many public health and basic clinic records improved. The findings suggest that a relatively brief intervention can improve village doctors’ use of and recording in the EHR if it is tailored to their needs and circumstances. It should be considered to support the implementation of the EHR in other disadvantaged rural areas in China.

## Competing interests

The authors have no competing interest in this research.

## Authors’ contributions

YZ from the School of Public Health, Nanchang University was responsible for the project, including project design, site implementation and supervision. He led the authorship of this paper. LY, LG and HP were involved in the evaluation data collection and analysis and critically reviewed and commented the draft paper. YJ coordinated the project support in Chongyi and critically reviewed and commented the draft paper. MH was advisor to the project during the design, implementation and evaluation stages. MH visited Chongyi and provided comment and advice and during visits of the researchers to Australia discussed the analysis and writing, reviewed and edited the paper. All authors read and approved the final manuscript.

## Pre-publication history

The pre-publication history for this paper can be accessed here:

http://www.biomedcentral.com/1472-6963/14/217/prepub
